# VIB5 database with accurate ab initio quantum chemical molecular potential energy surfaces

**DOI:** 10.1038/s41597-022-01185-w

**Published:** 2022-03-11

**Authors:** Lina Zhang, Shuang Zhang, Alec Owens, Sergei N. Yurchenko, Pavlo O. Dral

**Affiliations:** 1 State Key Laboratory of Physical Chemistry of Solid Surfaces, Fujian Provincial Key Laboratory of Theoretical and Computational Chemistry, Department of Chemistry, and College of Chemistry and Chemical Engineering, Xiamen University, Xiamen, 361005 China; 2 Department of Physics and Astronomy, University College London, Gower Street, WC1E 6BT London, United Kingdom

**Keywords:** Theoretical chemistry, Physical chemistry

## Abstract

High-level *ab initio* quantum chemical (QC) molecular potential energy surfaces (PESs) are crucial for accurately simulating molecular rotation-vibration spectra. Machine learning (ML) can help alleviate the cost of constructing such PESs, but requires access to the original *ab initio* PES data, namely potential energies computed on high-density grids of nuclear geometries. In this work, we present a new structured PES database called VIB5, which contains high-quality *ab initio* data on 5 small polyatomic molecules of astrophysical significance (CH_3_Cl, CH_4_, SiH_4_, CH_3_F, and NaOH). The VIB5 database is based on previously used PESs, which, however, are either publicly unavailable or lacking key information to make them suitable for ML applications. The VIB5 database provides tens of thousands of grid points for each molecule with theoretical best estimates of potential energies along with their constituent energy correction terms and a data-extraction script. In addition, new complementary QC calculations of energies and energy gradients have been performed to provide a consistent database, which, e.g., can be used for gradient-based ML methods.

## Abstract (summary)

**Table:** 

Measurement(s)	potential energy surfaces
Technology Type(s)	quantum chemistry computational methods

## Background & Summary

Many physical and chemical processes of molecular systems are governed by potential energy surfaces (PESs) that are functions of potential energy with respect to the molecular geometry defined by the nuclei^1^. Accurate *ab initio* quantum chemical (QC) molecular PESs are essential to predict and understand a multitude of physicochemical properties of interest such as reaction thermodynamics, kinetics^2^, and simulation of rovibrational spectra^3–5^. As for the latter, PESs of a number of different molecules have been constructed and used in variational nuclear motion calculations to provide accurate rotation-vibration-electronic line lists to aid the characterization of exoplanet atmospheres, amongst other applications^6–16^.

It is necessary to have a global PES covering all relevant regions of nuclear configurations allowing to simulate rotation-vibration (rovibrational) spectra approaching the coveted spectroscopic accuracy of 1 cm^−1^ in a broad range of temperatures. This can be achieved by defining the PES on a high-density grid of nuclear geometries with no holes and having the theoretical best estimate (TBE) of energies computed at a very high QC level of theory. The construction of an optimal grid usually involves many steps and human intervention, and often requires a staggeringly large number of grid points, e.g., ca. 100 thousand points even for a five-atom molecule such as methane^10^. The choice of QC level for TBE calculations is determined by the trade-off between accuracy and computational cost, but typically requires going well beyond the gold-standard^17–19^ CCSD(T)^17^/CBS (coupled cluster with single and double excitations and a perturbative treatment of triple excitations/complete basis set) limit and needs many QC corrections on top of it. Just to give a perspective, ca. 24 single processing unit (CPU)-hours are required for calculating TBE energy of each grid point of ~45 thousand methyl chloride (CH_3_Cl) geometries amounting to over 100 CPU-years when constructing its highly accurate ab initio PES^20^.

To reduce the high computational cost, machine learning (ML) has emerged as a powerful approach for constructing full-dimensional PESs^21–27^ and the resulting ML PESs can be used^22,24,28–35^ for performing vibrational calculations. In particular, substantial cost reduction can be achieved by calculating TBE energies only for a small number of existing grid points and then interpolating between them with ML^36^; such ML grids can be subsequently used for simulating rovibrational spectra with a relatively small loss of accuracy. Importantly, much larger savings in computational cost can be achieved^20^, when ML is applied to learn various QC corrections using a hierarchical ML (hML) scheme based on Δ-learning^37^ rather than to learn the TBE energy directly.

Despite all the above efforts in constructing highly accurate PESs, there is still room for improvement, e.g., via creating denser grids, using higher QC levels, and further development of ML approaches, all of which requires access to data. Unfortunately, the raw data containing geometries, TBEs and TBE constituent terms for many published studies is either missing or scattered. Thus, our data descriptor aims to organize these scattered data generated in the previous studies by some of us into a consolidated, structured PES database that we call VIB5. The VIB5 database contains five molecules CH_3_Cl^7,9,20^, CH_4_^10^, SiH_4_^8^, CH_3_F^12^, and NaOH^14^. The number of grid points ranges from 15 thousand to 100 thousand; altogether more than 300 thousand points (Table 1). In addition, it is also known that inclusion of the energy gradient information can significantly reduce the number of training points for ML, which is efficiently exploited in the gradient-based ML models^38,39^. Thus, for this database, we additionally calculate energies and energy gradients at two levels of theory, MP2/cc-pVTZ (second order Møller-Plesset perturbation theory/correlation-consistent triple-zeta basis set) and CCSD(T)/cc-pVQZ (correlation consistent quadruple-zeta basis set), and provide the HF (Hartree–Fock) energies calculated with the corresponding basis sets cc-pVTZ and cc-pVQZ.

**Table 1:** The number of grid points (grid size) for each molecule with references to original studies generating these grid points, theoretical best estimates (TBE), and TBE constituent terms.

Molecule	Grid size	Reference
CH_3_Cl	44819	^7,9,20^
CH_4_	97217^*a*^	^10^
SiH_4_	84002	^8^
CH_3_F	82653	^12^
NaOH	15901	^14^
Total: 5 molecules	324592^*a*^	

^*a*^The number of grid points is slightly smaller than that reported in the original publications as we found very few duplicates in the original data set. See section *Technical Validation*.

Our database is complementary to existing databases used for developing ML PES models. Some existing databases contain only energies for equilibrium geometries of various compounds calculated at different levels (from density functional theory [DFT] up to coupled-cluster approaches): QM7^40^, QM7b^41^, QM9^42^, revised QM9^43^, and ANI-1ccx^44^. Another database (ANI-1^45^) also contains energies at DFT for off-equilibrium geometries. Energies and energy gradients at DFT are available for equilibrium and off-equilibrium geometries of different molecules in the ANI-1x^44^ and QM7-X^46^ databases. The MD-17 dataset^38,39^ is a popular database with energies and energy gradients for geometries taken from MD trajectories of several small- to medium-sized molecules at DFT and for subset of points at CCSD(T) with different basis sets. PESs generated from MD are, however, likely to have limited coverage of high-energy geometries and many holes, making them inapplicable to some kinds of accurate simulations such as diffusion Monte Carlo calculations as was pointed out recently^47^. In contrast to these databases, our database provides reliable, global PESs with QC energies and energy gradients at different levels including very accurate TBEs of energies going beyond CCSD(T)/CBS, which can be used for ML models trained on data from several levels of theory, such as hML, Δ-learning, etc. Finally, our database comes with a convenient data-extraction script that can be used to pull the required information in a suitable format for, e.g., ML.

## Methods

### Grid points generation

For each molecule, we take grid points directly from the previous studies by some of the authors. Here we only describe in short how these grid points were generated for the sake of completeness. We refer the reader to the original publications cited for each molecule for further details (see Table 1).

#### CH_3_Cl

**Fig. 1:**
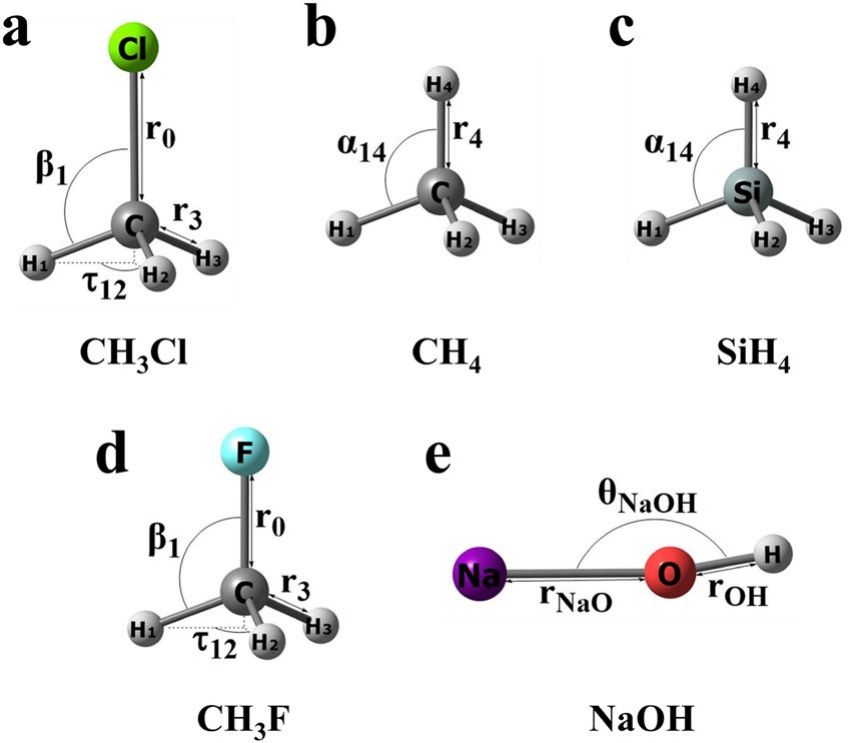
Definition of internal coordinates in each molecule. Internal coordinates of (**a**) CH_3_Cl; *r*_0_ is C–Cl bond length, *r*_*i*_ and *β*_*i*_ are C–H_*i*_ bond lengths and ∠(H_*i*_CCl) angles (*I* = 1, 2, 3), *τ*_*jk*_ are H_*j*_CClH_*k*_ dihedral angles (*jk* = 12, 13); only *r*_0_, *r*_3_, *β*_1_ and *τ*_12_ are shown; (**b**) CH_4_*; r*_*i*_ and *α*_*jk*_ are C–H_*i*_ bond lengths and ∠(H_*j*_CH_*k*_) angles (*i* = 1, 2, 3, 4; *jk* = 12, 13, 14, 23, 24); only *r*_4_ and *α*_14_ are shown; (**c**) SiH_4_*; r*_*i*_ and *α*_*jk*_ are Si–H_*i*_ bond lengths and ∠(H_*j*_SiH_*k*_) angles (*i* = 1, 2, 3, 4; *jk* = 12, 13, 14, 23, 24); only *r*_4_ and *α*_14_ are shown; (**d**) CH_3_F; *r*_0_ is C–F bond length, *r*_*i*_ and *β*_*i*_ are C–H_*i*_ bond lengths and ∠(H_*i*_CF) angles *(i* = 1, 2, 3), *τ*_*jk*_ are H_*j*_CFH_*k*_ dihedral angles (*jk* = 12, 13); only *r*_0_, *r*_3_, *β*_1_ and *τ*_12_ are shown; (**e**) NaOH; *r*_NaO_ and *r*_OH_ are Na–O and O–H bond lengths, *θ*_NaOH_ is ∠(NaOH) bond angle.

44819 grid points for CH_3_Cl were taken from Refs. ^7,9,20^. A Monte Carlo random energy-weighted sampling algorithm was applied to nine internal coordinates of CH_3_Cl: the C–Cl bond length *r*_0_; three C–H bond lengths *r*_1_, *r*_2_, and *r*_3_; three ∠(H_*i*_CCl) interbond angles *β*_1_, *β*_2_, and *β*_3_; and two dihedral angles *τ*_12_ and *τ*_13_ between adjacent planes containing H_*i*_CCl and H_*j*_CCl (Fig. 1a). This procedure led to geometries in the range 1.3 ≤ *r*_0_ ≤ 2.95 Å, 0.7 ≤ *r*_*i*_ ≤ 2.45 Å, 65 ≤ *β*_*i*_ ≤ 165° for *i* = 1, 2, 3 and 55 ≤ *τ*_*jk*_ ≤ 185° with *jk* = 12, 13. The grid also includes 1000 carefully chosen low-energy points to ensure an adequate description of the equilibrium region.

#### CH_4_

97271 grid points for CH_4_ were taken from ref. ^10^. The global grid was built in the same fashion as the grid was constructed for CH_3_Cl. Nine internal coordinates of CH_4_ are defined as follows: four C–H bond lengths *r*_1_, *r*_2_, *r*_3_ and *r*_4_; five∠(H_*j*_-C-H_*k*_) interbond angles *α*_12_, *α*_13_, *α*_14_, *α*_23_, and *α*_24_, where *j* and *k* label the respective hydrogen atoms (Fig. 1b). Then grid points are in the range 0.71 ≤ *ri* ≤ 2.60 Å for *i* = 1, 2, 3, 4 and 40 ≤ *α*_*jk*_ ≤ 140° with *jk* = 12, 13, 14, 23, 24.

#### SiH_4_

84002 grid points for SiH_4_ were taken from ref. ^8^. Nine internal coordinates of SiH_4_ are defined in the same way as CH_4_: four Si–H bond lengths *r*_1_, *r*_2_, *r*_3_ and *r*_4_; five∠(H_*j*_-Si-H_*k*_) interbond angles *α*_12_, *α*_13_, *α*_14_, *α*_23_, and *α*_24_, where *j* and *k* label the respective hydrogen atoms (Fig. 1c). Then geometries are in the range 0.98 ≤ *ri* ≤ 2.95 Å for *i* = 1, 2, 3, 4 and 40 ≤ *α*_*jk*_ ≤ 140° with *jk* = 12, 13, 14, 23, 24.

#### CH_3_F

82653 grid points for CH_3_F were taken from ref. ^12^. Nine internal coordinates of CH_3_F are defined in the same way as CH_3_Cl: the C–F bond length *r*_0_; three C–H bond lengths *r*_1_, *r*_2_, and *r*_3_; three ∠(H_*i*_CF) interbond angles *β*_1_, *β*_2_, and *β*_3_; and two dihedral angles *τ*_12_ and *τ*_13_ between adjacent planes containing H_*i*_CF and H_*j*_CF (Fig. 1d). This procedure led to geometries in the range 1.005 ≤ *r*_0_ ≤ 2.555 Å, 0.705 ≤ *r*_*i*_ ≤ 2.695 Å, 45.5 ≤ *β*_*i*_ ≤ 169.5° for *i* = 1, 2, 3 and 40.5 ≤ *τ*_*jk*_ ≤ 189.5° with *jk* = 12, 13.

#### NaOH

15901 grid points for NaOH were taken from ref. ^14^. Grid points were generated randomly with a dense distribution around the equilibrium region. Three internal coordinates of NaOH are defined as follows: the Na–O bond length *r*_NaO_, the O–H bond length *r*_OH_, and the interbond angle ∠(NaOH) (Fig. 1e). This procedure led to geometries in the range 1.435 ≤ *r*_NaO_ ≤ 4.400 Å, 0.690 ≤ *r*_OH_ ≤ 1.680 Å, and 40 ≤ ∠(NaOH) ≤ 180°.

### Theoretical best estimates and constituent terms

For each molecule, we take the TBEs and energy corrections directly from the previous studies by some of us. Here we only briefly introduce how these calculations were performed. We refer the reader to the original publications cited for each molecule for details (see Table 1). TBE is obtained through the sum of many constituent terms: *E*_CBS_, ∆*E*_CV_, ∆*E*_HO_, ∆*E*_SR_, and, for most molecules, ∆*E*_DBOC_. *E*_CBS_ means the energy at the complete basis set (CBS) limit. ∆*E*_CV_ refers to the core-valence (CV) electron correlation energy correction. ∆*E*_HO_ refers to the energy correction accounted for by the higher-order (HO) coupled cluster terms and ∆*E*_SR_ shows scalar relativistic (SR) effects. ∆*E*_DBOC_ means the diagonal Born–Oppenheimer correction and was calculated for CH_3_Cl, CH_4_, CH_3_F, and NaOH, but not for SiH_4_ due to the little effect of ∆*E*_DBOC_ on the vibrational energy levels of this molecule.

The constituent terms were not calculated at the same level of theory across all molecules in the data set. The computational details of five TBE constituent terms (*E*_CBS_, ∆*E*_CV_, ∆*E*_HO_, ∆*E*_SR_, and ∆*E*_DBOC_) for 5 molecules are shown below and summarized in the Table 2.

**Table 2:** The comparative table of the computational details behind the calculations of the constituent terms of theoretical best estimates for five molecules of the VIB5 database.

Molecule	*E*_CBS_	∆*E*_CV_	∆*E*_HO_	∆*E*_SR_	∆*E*_DBOC_
CH_3_Cl	Software: MOLPRO2012	The basis set: cc-pCVQZ-F12; Slater geminal exponent value *β* = 1.5 *a*_0_^−1^; all-electron calculations kept the 1*s* orbital of Cl frozen; Software: MOLPRO2012	Levels of theory: CCSD(T), CCSDT, and CCSDT(Q); Basis sets for the full triples and the perturbative quadruples calculations are aug-cc-pVTZ(+d for Cl) and aug-cc-pVDZ(+d for Cl), respectively.	Method: one-electron mass velocity and Darwin (MVD1) terms from the Breit–Pauli Hamiltonian in first-order perturbation theory; All electrons correlated (except for the 1*s* of Cl); CCSD(T)/aug-cc-pCVTZ(+d for Cl). Software: CFOUR	The 1*s* orbital of Cl is frozen and all other electrons are correlated; basis set: aug-cc-pCVTZ (+d for Cl)
CH_4_	Software: MOLPRO2012	The basis set: cc-pCVTZ-F12; Slater geminal exponent value *β* = 1.4 *a*_0_^−1^; No frozen orbital; Software: MOLPRO2012	Levels of theory: CCSD(T), CCSDT, and CCSDT(Q); Basis sets for the full triples and the perturbative quadruples calculations are cc-pVTZ and cc-pVDZ, respectively.	Method: the second-order Douglas–Kroll–Hess approach; frozen core approximation; CCSD(T)*/*cc-pVQZ-DK. Software: MOLPRO2012	All electrons are correlated; basis set: aug-cc-pCVDZ
SiH_4_	Software: MOLPRO2012	The basis set: cc-pCVTZ-F12; Slater geminal exponent value *β* = 1.4 *a*_0_^−1^; all-electron calculations kept the 1*s* orbital of Si frozen; Software: MOLPRO2012	Levels of theory: CCSD(T), CCSDT, and CCSDT(Q); basis sets for the full triples and the perturbative quadruples calculations are cc-pVTZ(+d for Si) and cc-pVDZ(+d for Si), respectively.	Method: the second-order Douglas-Kroll-Hess approach; frozen core approximation; CCSD(T)*/*cc-pVQZ-DK. Software: MOLPRO2012	The correction was not included.
CH_3_F	Software: MOLPRO2012	The basis set: cc-pCVTZ-F12; Slater geminal exponent value *β* = 1.4 *a*_0_^−1^; no frozen orbital; Software: MOLPRO2012	Levels of theory: CCSD(T), CCSDT, and CCSDT(Q); basis sets for the full triples and the perturbative quadruples calculations are cc-pVTZ and cc-pVDZ, respectively.	Method: the second-order Douglas–Kroll–Hess approach; frozen core approximation; CCSD(T)/ cc-pVQZ-DK. Software: MOLPRO2012	All electrons are correlated; basis set: aug-cc-pCVDZ
NaOH	Software: MOLPRO2015	The basis set: cc-pCVTZ-F12; Slater geminal exponent value *β* = 1.4 *a*_0_^−1^; all-electron calculations kept the 1*s* orbital of sodium frozen; Software: MOLPRO2015	Levels of theory: CCSD(T) and CCSDT; basis set: cc-pVTZ(+d for Na).	Method: the second-order Douglas–Kroll–Hess approach; frozen core approximation; CCSD(T)/cc-pVQZ-DK. Software: MOLPRO2015	The 1*s* orbital of Na is frozen and all other electrons are correlated; basis set: aug-cc-pCVDZ(+d for Na)

This table mainly emphasizes differences for each molecule, rather than giving the full description of computational details.

#### E_CBS_

To extrapolate the energy to the CBS limit, the parameterized, two-point formula^48^ $\left({E}_{CBS}^{C}=\left({E}_{n+1}-{E}_{n}\right){F}_{n+1}^{C}+{E}_{n}\right)$ was used. In this process, the method CCSD(T)-F12b^49^ and two basis sets cc-pVTZ-F12 and cc-pVQZ-F12^50^ were chosen. When performing calculations, the frozen core approximation was adopted and the diagonal fixed amplitude ansatz 3C(FIX)^51^ with a Slater geminal exponent value^48^ of *β* = 1.0 *a*_0_^−1^ were employed. As for the auxiliary basis sets (ABS), the resolution of the identity OptRI^52^ basis and cc-pV5Z/JKFIT^53^ and aug-cc-pwCV5Z/MP2FIT^54^ basis sets for density fitting were used for all 5 molecules. These calculations were carried out with either MOLPRO2012^55^ (CH_3_Cl, CH_4_, SiH_4_, CH_3_F) or MOLPRO2015^55,56^ (NaOH). As for the coefficients ${F}_{n+1}^{C}$ in this two-point formula, *F*^CCSD-F12b^ = 1.363388 and *F*^(T)^ = 1.769474^48^ were used for all molecules. The extrapolation was not applied to the Hartree–Fock (HF) energy and the HF + CABS (complementary auxiliary basis set) singles correction^49^ calculated with the cc-pVQZ-F12 basis set was used.

#### ∆E_CV_

∆*E*_CV_ was computed at CCSD(T)-F12b/cc-pCVQZ-F12^57^ for CH_3_Cl and at CCSD(T)-F12b/cc-pCVTZ-F12^57^ for the other 4 molecules (CH_4_, SiH_4_, CH_3_F, NaOH). The same ansatz and ABS used for *E*_CBS_ were employed for calculating ∆*E*_CV_ but the Slater geminal exponent value was changed: *β* = 1.5 *a*_0_^−1^ for CH_3_Cl and *β* = 1.4 *a*_0_^−1^ for the other 4 molecules. For this term, all-electron calculations were adopted, but with the 1*s* orbital of Cl frozen for CH_3_Cl, the 1*s* orbital of Si frozen for SiH_4_, and the 1*s* orbital of Na frozen for NaOH. There is no frozen orbital in all-electron calculations for CH_4_ and CH_3_F. As for the software used, see the above *E*_CBS_ part.

#### ∆E_HO_

To obtain ∆*E*_HO_, the hierarchy of coupled cluster methods was used. ∆*E*_HO_ = *E*_CCSDT_ − *E*_CCSD(T)_ for NaOH, while ∆*E*_HO_ = ∆*E*_T_ + ∆*E*_(Q)_ for other 4 molecules (CH_3_Cl, CH_4_, SiH_4_, CH_3_F) with ∆*E*_T_ = *E*_CCSDT_ − *E*_CCSD(T)_ for full triples contribution and ∆*E*_(Q)_ = *E*_CCSDT(Q)_ − *E*_CCSDT_ for perturbative quadruples contribution. The frozen core approximation was employed in the calculations. Thus, energy calculations at CCSD(T) and CCSDT were performed for NaOH, while energy calculations at CCSD(T), CCSDT, and CCSDT(Q) levels of theory were performed for other 4 molecules. All of these calculations were carried out through the general coupled cluster approach^58,59^ implemented in the MRCC code (www.mrcc.hu)^60^ interfaced to CFOUR (www.cfour.de)^61^. As for the basis set, aug-cc-pVTZ(+d for Cl)^62–65^ & aug-cc-pVDZ(+d for Cl), cc-pVTZ^62^ & cc-pVDZ, cc-pVTZ(+d for Si)^62–65^ & cc-pVDZ(+d for Si), and cc-pVTZ^62^ & cc-pVDZ for full triples and the perturbative quadruples of CH_3_Cl, CH_4_, SiH_4_, and CH_3_F. For NaOH, cc-pVTZ(+d for Na)^62,66^ were used for CCSD(T) and CCSDT calculations.

#### ∆E_SR_

∆*E*_SR_ was calculated by using either one-electron mass velocity and Darwin (MVD1) terms from the Breit–Pauli Hamiltonian in first-order perturbation theory^67^ or the second-order Douglas–Kroll–Hess approach^68,69^. The former method was used for CH_3_Cl and the latter method was used for the other 4 molecules (CH_4_, SiH_4_, CH_3_F, and NaOH). All-electron calculations (except for the 1*s* orbital of Cl) was adopted for CH_3_Cl while the frozen core approximation was employed for the other 5 molecules. Calculations were performed at CCSD(T)/aug-cc-pCVTZ(+d for Cl)^70,71^ using the MVD1 approach^72^ implemented in CFOUR for CH_3_Cl and at CCSD(T)/cc-pVQZ-DK^73^ using MOLPRO (software versions the same as mentioned in the above *E*_CBS_ part) for other 4 molecules.

#### ∆E_DBOC_

∆*E*_DBOC_ was computed using the CCSD method^74^ as implemented in CFOUR. This correction was not included for SiH_4_. For this term, all-electron calculations were adopted, but with the 1*s* orbital of Cl frozen for CH_3_Cl, all electrons correlated for CH_4_ and CH_3_F, and the 1*s* orbital of Na frozen for NaOH. As for the basis set, calculations were performed at aug-cc-pCVTZ (+d for Cl) for CH_3_Cl, aug-cc-pCVDZ for CH_4_, aug-cc-pCVDZ for CH_3_F, and aug-cc-pCVDZ(+d for Na) for NaOH.

### Complementary energy and gradient calculations

**Fig. 2:**
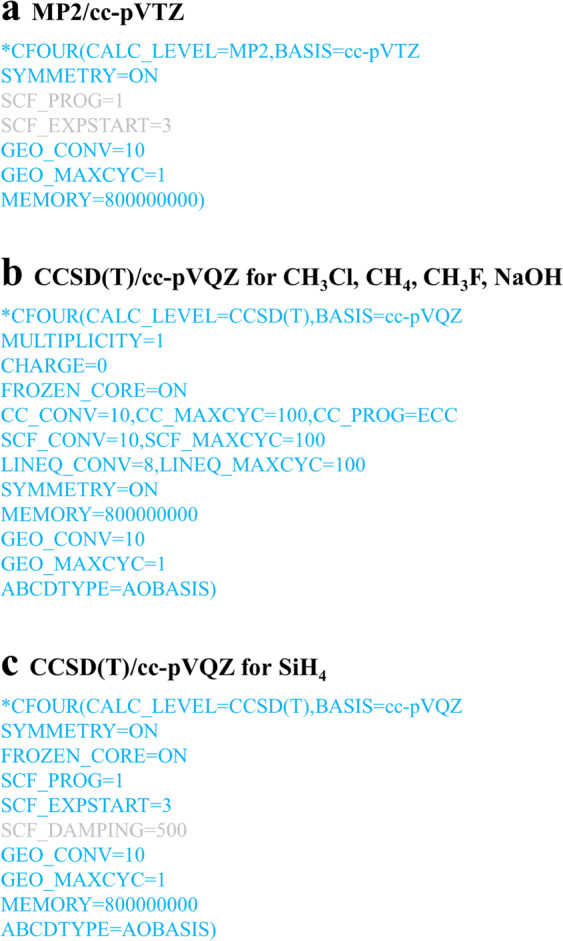
Typical CFOUR input options for (**a**) MP2/cc-pVTZ, (**b**) CCSD(T)/cc-pVQZ for CH_3_Cl, CH_4_, CH_3_F, NaOH and (**c**) CCSD(T)/cc-pVQZ for SiH_4_. The blue options were used for most cases and the light grey options are examples of options used to improve SCF convergence only for some geometries.

All complementary *ab initio* QC energy and gradient calculations for a total of 324592 grid points were performed with two levels of theory: MP2^75,76^/cc-pVTZ^62,64,66^ and CCSD(T)^17,77,78^/cc-pVQZ^62,64,66^ using the CFOUR program package (Versions 1.0 and 2.1^61^; we use CFOUR V2.1 to perform calculations for some grid points in CH_3_Cl and NaOH that converge to high energy solutions); see Fig. 2 for the CFOUR input options. In the MP2/cc-pVTZ calculations, we use the default option FROZEN_CORE = OFF so that all electrons and all orbitals are correlated. In the CCSD(T)/cc-pVQZ calculations, the option FROZEN_CORE = ON is used for all molecules to allow valence electrons correlation alone. For CH_3_Cl, CH_4_, CH_3_F and NaOH, SCF_CONV = 10, CC_CONV = 10 and LINEQ_CONV = 8 are set to specify the convergence criterion for the HF-SCF, CC amplitude and linear equations and CC_PROG = ECC is set to specify that the CC program we used is ECC. For SiH_4_, we adopted CFOUR default options SCF_CONV = 7, CC_CONV = 7, LINEQ_CONV = 7 and CC_PROG = VCC. We use GEO_MAXCYC = 1 option to set the maximum number of geometry optimization iterations to one to obtain the gradient information of the current nuclear configuration. From these calculations we also extracted HF energies calculated with the corresponding basis sets cc-pVTZ and cc-pVQZ. In addition, for CH_3_Cl we include MP2/aug-cc-pVQZ energies calculated using MOLPRO2012^55^ as reported in ref. ^20^.

## Data Records

**Fig. 3:**
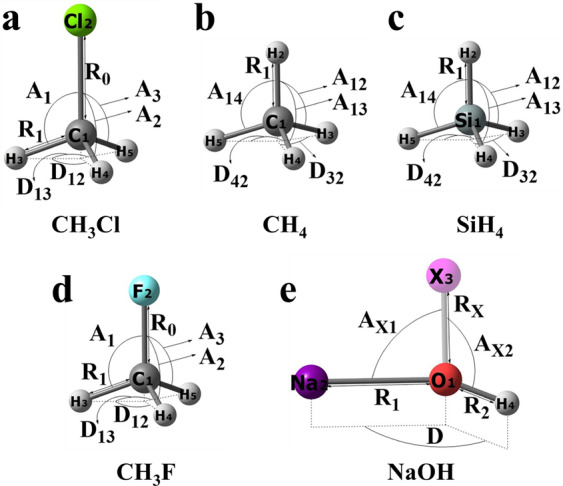
Definition of internal coordinates for each molecule used in the database file VIB5.json and in the complimentary calculations. Internal coordinates of (**a**) CH_3_Cl; R_0_ is C–Cl bond length, R_*i*_ and A_*i*_ are C–H_*i*+2_ bond lengths and ∠(H_*i*+2_CCl) angles (*i* = 1, 2, 3), D_*jk*_ are H_*j*+2_CClH_*k*+2_ dihedral angles (*jk* = 12, 13); only R_0_, R_1_, A_1_, A_2_, A_3_, D_12_, and D_13_ are shown; (**b**) CH_4_; R_*i*_ and A_1*j*_ are C–H_*i*+1_ bond lengths and ∠(H_2_CH_*j*+1_) angles (*i* = 1, 2, 3, 4; *j* = 2, 3, 4), D_*k*2_ are H_*k*+1_CH_2_H_3_ dihedral angles (*k* = 3, 4); only R_1_, A_12_, A_13_, A_14_, D_32_, and D_42_ are shown; (**c**) SiH_4_; R_*i*_ and A_1*j*_ are Si–H_*i*+1_ bond lengths and ∠(H_2_SiH_*j*+1_) angles (*i* = 1, 2, 3, 4; *j* = 2, 3, 4), D_*k*2_ are H_*k*+1_SiH_2_H_3_ dihedral angles (*k* = 3, 4); only R_1_, A_12_, A_13_, A_14_, D_32_, and D_42_ are shown; (**d**) CH_3_F; R_0_ is C–F bond length, R_*i*_ and A_*i*_ are C–H_*i*+2_ bond lengths and ∠(H_*i*+2_CF) angles (*i* = 1, 2, 3), D_*jk*_ are H_*j*+2_CFH_*k*+2_ dihedral angles (*jk* = 12, 13); only R_0_, R_1_, A_1_, A_2_, A_3_, D_12_, and D_13_ are shown; (**e**) NaOH; R_1_ and R_2_ are Na–O and H–O bond lengths, R_X_ is O–X bond length, A_X1_ and A_X2_ are ∠(XONa) and ∠(XOH) angles, and D is NaXOH dihedral angle. X is a dummy atom.

All data of 5 molecules are stored as a database in JSON format in the file named VIB5.json available for download from 10.6084/m9.figshare.16903288^79^. The first level of the database contains an item corresponding to each molecule in the order of CH_3_Cl, CH_4_, SiH_4_, CH_3_F, and NaOH. For each molecule, at the next level of the database, chemical formula, chemical name, number of atoms, list of nuclear charges in the same order as they appear in the items with nuclear coordinates are given at first, then the description of properties available for grid points (property type, levels of theory, units) is provided. Finally, the items for each grid point are given containing nuclear positions in both Cartesian and internal coordinates, and the values of properties (energies and energy gradients at different levels of theory, i.e., TBE, TBE constituent terms, complementary data). The JSON keys of items available for each grid point are listed in Table 3 with the brief description and units. The geometry configuration in Cartesian coordinates and in internal coordinates of each grid point for each molecule can be accessed by the “XYZ” key and the “INT” key, respectively. Definition of internal coordinates used in the database is shown in Fig. 3. The “HF-TZ”, “HF-QC”, “MP2”, “CCSD-T”, and “TBE” keys can be selected separately to obtain the energy of each grid point at HF/cc-pVTZ, HF/cc-pVQZ, MP2/cc-pVTZ, CCSD(T)/cc-pVQZ, and TBE, respectively. This database also provides the energy gradients in Cartesian coordinates and internal coordinates at MP2/cc-pVTZ and CCSD(T)/cc-pVQZ theory levels, which can be accessed through “MP2_grad_xyz”, “MP2_grad_int”, “CCSD-T_grad_xyz”, and “CCSD-T_grad_int” keys. See Table 3 for the summary and the keys of other properties.

**Table 3:** Layout of the VIB5.json file containing the VIB5 database.

No.	Key	Description	Units
1	XYZ	Nuclear positions in Cartesian coordinates	Å
2	INT	Nuclear positions in internal coordinates	Å; degree
3	HF-TZ	Total energy at HF/cc-pVTZ	Hartree
4	HF-QZ	Total energy at HF/cc-pVQZ	Hartree
5	MP2	Total energy at MP2/cc-pVTZ	Hartree
6	CCSD-T	Total energy at CCSD(T)/cc-pVQZ	Hartree
7	TBE	Theoretical best estimate of ab initio deformation energies	cm^−1^
8	MP2_grad_xyz	Energy gradient in Cartesian coordinates at MP2/cc-pVTZ	Hartree/Å
9	MP2_grad_int	Energy gradient in internal coordinates at MP2/cc-pVTZ	Hartree/Å; Hartree/degree
10	CCSD-T_grad_xyz	Energy gradient in Cartesian coordinates at CCSD(T)/cc-pVQZ	Hartree/Å
11	CCSD-T_grad_int	Energy gradient in internal coordinates at CCSD(T)/cc-pVQZ	Hartree/Å; Hartree/degree
12	CBS	Deformation energies at CCSD(T)-F12b/CBS	cm^−1^
13	VTZ	Deformation energies at CCSD(T)-F12b/cc-pVTZ-F12 (only for CH_3_Cl molecule)	cm^−1^
14	VQZ	Deformation energies at CCSD(T)-F12b/cc-pVQZ-F12 (only for CH_3_Cl molecule)	cm^−1^
15	CV	Deformation energy corrections to account for core-valence electron correlation	cm^−1^
16	HO	Deformation higher-order coupled cluster terms beyond perturbative triples	cm^−1^
17	SR	Deformation scalar relativistic (SR) effects	cm^−1^
18	DBOC	Deformation diagonal Born–Oppenheimer corrections (only for CH_3_Cl, CH_4_, CH_3_F, and NaOH molecules)	cm^−1^
19	MP2-aQZ	Deformation energies at MP2/aug-cc-pVQZ (only for CH_3_Cl molecule)	cm^−1^

## Technical Validation

The TBE values and TBE constituent terms were validated by calculating rovibrational spectra and comparing them to experiment in the original peer-reviewed publications cited in the *Methods* section and Table 1. In brief, rovibrational energy levels were computed by fitting analytical expression for PES and performing with it variational calculations using the nuclear motion program TROVE^80^. Then the resulting line list of rovibrational energy levels was compared to experimental values (when available) to validate the accuracy of the underlying PES. The new complementary data we have calculated here was validated by making sure that all calculations fully converged. After the database was constructed, we performed additional checks for repeated geometries, which identified grid points with the same geometrical parameters in the CH_4_ grid points. We removed such duplicates from the database, which leads to a slightly reduced number of points (97217) compared to the numbers reported in the original publications (97271). This pruned grid is used as our final database.

## Usage Notes

We provide a Python script extraction_data.py that can be used to pull the data of interest from the VIB5.json (Box 1). It is provided together with the database file from 10.6084/m9.figshare.16903288^79^.

Box 1 Using extraction_data.py script to extract required data: an example of extracting CCSD(T)/CBS and CCSD(T)/cc-pVQZ energies and Cartesian geometries for NaOH. The *.dat files contain energies and *.xyz files contain XYZ geometries in the same order as in the database. The user can run python3 extraction_data.py -h command to see more options.

example$ ls

VIB5.json extraction_data.py

example$ python3 ./extraction_data.py --mols NaOH --energy CBS,CCSD-T -xyz

example$ ls

NaOH_CBS.dat NaOH_CCSD-T.dat NaOH.xyz VIB5.json extraction_data.py

example$ head -n 10 *.dat

==> NaOH_CBS.dat <==

59.280650000000

59.574700000000

59.558345000000

47.465761000000

64.042693000000

59.852814000000

60.391809000000

61.782135000000

33.479406000000

83.271969000000

==> NaOH_CCSD-T.dat <==

-237.644636975222

-237.644635947086

-237.644635792937

-237.644692089151

-237.644614779690

-237.644634762797

-237.644632245341

-237.644626330200

-237.644757449209

-237.644525233060

example$ head -n 10 *.xyz

3

O                   0.00000000         0.00000000        1.08916506

Na                 0.00000000         0.00000000        -0.84719335

H                   0.00000000         0.00000000        2.03971526

3

O                   0.00000000         0.00000000        1.08917892

Na                 0.00000000         0.00000000        -0.84717949

H                   0.00000000         0.00000000        2.03917912

## Data Availability

All the data generated at the MP2/cc-pVTZ and the CCSD(T)/cc-pVQZ levels of theory were performed with the CFOUR software package. TBE and other data were obtained using various software packages (MOLPRO, CFOUR, MRCC) as described in the Methods section.
